# Genome Sequences of Newly Isolated Mycobacteriophages Forming Cluster S

**DOI:** 10.1128/genomeA.00933-16

**Published:** 2016-09-29

**Authors:** Monique L. Mills, Judd Bragg, Asri Bruce, Ari Dehn, Jordan Drouin, Morgan Hefner, Dylan Katon, Dustin McHugh, Franck Zeba, Charles A. Bowman, Steven G. Cresawn, Deborah Jacobs-Sera, Daniel A. Russell, Welkin H. Pope, Graham F. Hatfull, David A. Dunbar, Gerard P. Zegers, Shallee T. Page

**Affiliations:** a Division of Environmental and Biological Sciences, University of Maine at Machias, Machias, Maine, USA; b Department of Biological Sciences, University of Pittsburgh, Pittsburgh, Pennsylvania, USA; c James Madison University, Harrisonburg, Virginia, USA; d Cabrini College, Radnor, Pennsylvania, USA

## Abstract

We describe the genomes of two mycobacteriophages, MosMoris and Gattaca, newly isolated on *Mycobacterium smegmatis*. The two phages are very similar to each other, differing in 61 single nucleotide polymorphisms and six small insertion/deletions. Both have extensive nucleotide sequence similarity to mycobacteriophage Marvin and together form cluster S.

## GENOME ANNOUNCEMENT

Mycobacteriophages are viruses that specifically infect a mycobacterial host. *Mycobacterium smegmatis* is currently a host to >6,900 phages (1). Phage populations are numerous and diverse: it is estimated that 10^31^ different particles exist (2, 3). Based on shared gene content, these phages are divided into 26 clusters, with singletons that do not belong to any clusters. The genomes in this report establish cluster S, one of the smaller clusters, making up 0.2% of current known phages and currently containing no subclusters (1, 2). Marvin was the first discovered cluster S phage and remained a singleton until the discovery of two similar phages: MosMoris and Gattaca (see GenBank nucleotide sequence accession numbers in Table 1).

**TABLE 1:** Data for three S cluster phages

Phage	Yr of isolation	GenBank accession no.	Length (bp)	G+C (%)	No. of ORFs
Gattaca	2014	KX159477	65,237	63.3	115
Marvin	2009	JF704100	65,100	63.4	107
MosMoris	2012	KJ538721	65,243	63.4	111

One of the phages, Marvin, was isolated in Radnor, PA; the other two, MosMoris and Gattaca, were isolated in Milbridge, ME (1), from soil samples in different years by direct plating. Once isolated, the phages form plaques on a lawn of *M. smegmatis* mc^2^ 155. Plaques were picked, purified, and amplified, and the DNA was extracted. Marvin, MosMoris, and Gattaca were sequenced at the Joint Genome Institute (Sanger sequencing) and the Pittsburgh Bacteriophage Institute (Ion Torrent and Illumina sequencing), respectively.

After sequencing, reads were assembled using Newbler and Consed. The DNA Master software (http://cobamide2.bio.pitt.edu) was used to annotate the genomes with an integrated approach using Glimmer, GeneMark, BLAST, and Shine-Dalgarno positioned weight scores. Phamerator (4) and HHpred (5) were used to predict the function of hypothetical proteins.

These cluster S mycobacteriophages display a morphotype of *Siphoviridae* and have an average G+C content of 64.3% (1). The average length of members of cluster S was 65,193 bp (1), and they are 99% identical to each other (2). Each phage in the cluster has a 3′ sticky overhang of 11 bp that contains a sequence of GCGCGCAGCGC (1).

The three genomes have an average of 111 open reading frames (ORFs) (Table 1). All of the genes are forward-transcribed genes except for 10 to 11 ORFs near gp100 and two single leftwards-transcribed genes in the left parts of the genomes: a DNA methylase and gene of unknown function. Many of the genes, particularly in the rightmost parts of the genomes, are of unknown function. However, the following were identified by homology: terminase, tape measure protein, minor tail protein, lysin A, lysin B, holin, WhiB, exonuclease, hydrolase, methyltransferase, galactosyl transferase, and a glycosyl transferase. The genes are syntenic and tightly packed, except for the region where the direction of transcription changes. MosMoris and Gattaca both contain insertions of an HNH endonuclease in the minor tail genes that is absent in Marvin. In addition, Marvin has a putative insertion with low coding potential near 9,500 bp that is not seen in the other two cluster members.

The S cluster’s closest relationships by nucleotide similarity map are two singletons, Wildcat and Sparky (2), two phages with low similarity to other phages. The closest clusters to the S cluster are the M and T clusters.

### Accession number(s).

The whole-genome sequences have been deposited at GenBank under the accession numbers listed in Table 1.

## References

[1] Howard Hughes Medical Institute 2015 The actinobacteriophage database. Howard Hughes Medical Institute, Chevy Chase, MD http://phagesdb.org/.

[2] Pope WH, Bowman CA, Russell DA, Jacobs-Sera D, Asai DJ, Cresawn SG, Jacobs WR, Hendrix RW, Lawrence JG, Hatfull GF 2015 Whole genome comparison of a large collection of mycobacteriophages reveals a continuum of phage genetic diversity. Elife 4:e06416. doi:10.7554/eLife.06416. 25919952 PMC4408529

[3] Hatfull G Mycobacteriophages: windows into tuberculosis. PLoS Pathog 10:e1003953. 24651299 10.1371/journal.ppat.1003953 PMC3961340

[4] Söding J, Biegert A, Lupas AN 2005 The HHpred interactive server for protein homology detection and structure prediction. doi:10.1093/nar/gki408. PMC1160169 15980461

[5] Cresawn SG, Bogel M, Day N, Jacobs-Sera D, Hendrix RW, Hatfull GF 2011 Phamerator: a bioinformatic tool for comparative bacteriophage genomics. BMC bioinformatics. doi:10.1186/1471-2105-12-395. PMC3233612 21991981

